# A medical big data access control model based on smart contracts and risk in the blockchain environment

**DOI:** 10.3389/fpubh.2024.1358184

**Published:** 2024-03-28

**Authors:** Xuetao Pu, Rong Jiang, Zhiming Song, Zhihong Liang, Liang Yang

**Affiliations:** ^1^Faculty of Information Engineering and Automation, Kunming University of Science and Technology, Kunming, China; ^2^Yunnan Key Laboratory of Service Computing, Yunnan University of Finance and Economics, Kunming, China; ^3^Institute of Intelligence Applications, Yunnan University of Finance and Economics, Kunming, China; ^4^Institute of Big Data and Artificial Intelligence, Southwest Forestry University, Kunming, China

**Keywords:** medical big data, access control, smart contracts, risk, blockchain

## Abstract

The rapid development of the Hospital Information System has significantly enhanced the convenience of medical research and the management of medical information. However, the internal misuse and privacy leakage of medical big data are critical issues that need to be addressed in the process of medical research and information management. Access control serves as a method to prevent data misuse and privacy leakage. Nevertheless, traditional access control methods, limited by their single usage scenario and susceptibility to single point failures, fail to adapt to the polymorphic, real-time, and sensitive characteristics of medical big data scenarios. This paper proposes a smart contracts and risk-based access control model (SCR-BAC). This model integrates smart contracts with traditional risk-based access control and deploys risk-based access control policies in the form of smart contracts into the blockchain, thereby ensuring the protection of medical data. The model categorizes risk into historical and current risk, quantifies the historical risk based on the time decay factor and the doctor’s historical behavior, and updates the doctor’s composite risk value in real time. The access control policy, based on the comprehensive risk, is deployed into the blockchain in the form of a smart contract. The distributed nature of the blockchain is utilized to automatically enforce access control, thereby resolving the issue of single point failures. Simulation experiments demonstrate that the access control model proposed in this paper effectively curbs the access behavior of malicious doctors to a certain extent and imposes a limiting effect on the internal abuse and privacy leakage of medical big data.

## 1 Introduction

Hospital Information System (HIS) is a medical information management system that leverages computer and information management technologies, with a focus on service and clinical applications (1). The HIS plays a pivotal role in modern healthcare. In recent years, the rapid development of the HIS has led to an accumulation of substantial data in the healthcare field. These data, generated through medical records, regulatory requirements, and medical research, encompass a wide range of areas, including administrative claims, electronic medical records, disease surveillance, medical imaging, laboratory tests, among others (2). This accumulation has given rise to the concept and development of medical big data, a large-scale collection of data within the healthcare domain that is collected, stored, and analyzed. Much like general big data, medical big data is characterized by its large volume, velocity, and diversity (3). Through the integration and analysis of these data using information technology tools and analytics, healthcare organizations, doctors, and researchers can gain a better understanding of disease pathogenesis, develop more effective treatment plans, and enhance healthcare services and preventive measures. The utilization of medical big data is anticipated to aid in the advancement of personalized medicine, precision medicine, and health management, thereby improving the quality and efficiency of healthcare.

While the potential value of healthcare data is being harnessed to improve the quality of healthcare services, enhance treatment outcomes, and reduce healthcare costs, issues such as misuse of healthcare data and privacy leakage have emerged. For instance, in February 2017, two staff members of the Shanghai Center for Disease Control and Prevention (CDC) and the Huangpu District CDC illicitly appropriated 200,000 pieces of information on newborn babies and sold them to infant healthcare product operators. In September 2022, an employee of Kaiser Permanente unauthorizedly accessed a segment of a patient’s medical record in the Mid-Atlantic region. This incident led to the exposure of substantial basic and medical information of the patient. Given the specificity and sensitivity of healthcare data, these privacy breaches, driven by various illicit interests, have inflicted substantial losses on healthcare systems and patients. Currently, the privacy leakage of medical data has become a pressing issue in the healthcare industry.

As for the internal leakage of medical data, the principal method to prevent errors and over-authorization is the establishment of an access control system. Traditional access control methods such as Discretionary Access Control (DAC) (4), Mandatory Access Control (MAC) (5), and Role-Based Access Control (RBAC) (6) are characterized by their static and explicit authorization rules. However, these methods are unable to adapt to the dynamic and real-time characteristics of medical big data scenarios (7), and they also exacerbate the workload of information system administrators. Risk-Adaptive Access Control (RAdAC) (8) is an access control method that incorporates risk into the policy to facilitate the dynamic adjustment of subject privileges. Despite the fact that the dynamic adjustment of subject privileges can be well-suited to medical big data scenarios, risk-based access control is fundamentally a centralized authorization scheme. This centralization is susceptible to causing a ‘single point of failure’, which can impact the normal operation of the system.

Blockchain is a decentralized Distributed Ledger Technology (DLT) that offers advantages like immutability, tamper-proof, traceability, and authentication (9). The access history and authorization rules in the HIS can be permanently stored in the blockchain. Smart contracts, computer programs stored in the blockchain system, record the result of any execution of the program in the blockchain system (10). In recent years, the application of smart contracts to blockchain by numerous scholars has endowed blockchain with the capability to implement distributed applications (11). Consequently, blockchain is considered a traceable and verifiable platform in distributed access control, effectively addressing issues such as the single point of failure inherent in traditional centralized access control (12).

In this study, we explore a risk-adaptive access control model tailored for medical big data and implement the access control policy as a smart contract within a blockchain. We introduce a smart contracts and risk-based access control model (SCR-BAC). Our main contributions are summarized as follows. The SCR-BAC is distributed, dynamic, and adaptive, making it well-suited to complex medical big data access control scenarios.

1. We introduce a smart contract and risk-based access control model. Specifically, we modify the traditional risk-based access control to cater to distributed services within the context of healthcare big data. This adaptation not only mitigates the ‘single point of failure’ problem inherent in traditional RAdAC to a certain extent but also enhances the efficiency of traditional RAdAC.
2. We propose a method to quantify risk based on both current and historical behavior, taking into account the impact of historical behavior. Specifically, we utilize the access history in the blockchain to evaluate a doctor’s historical risk. We then quantify the current risk using the actual offset metrics of the doctor’s medical record choices and work goals. Furthermore, we employ a time decay factor to articulate the influence of historical behavior on the present situation.
3. Given the complex and dynamic nature of medical big data scenarios, we have designed a risk management mechanism to implement dynamic access control. This mechanism utilizes the risk averages of malicious doctors over a certain period to compute risk thresholds. This approach aids in distinguishing between honest and malicious doctors more effectively, thereby enhancing the accuracy of the system.

The remainder of this paper is structured as follows: The ‘Related work’ section provides an overview of the current state of research on access control. The ‘Access control model’ section offers a detailed description of the access control model proposed in this paper. The ‘Experimental and evaluation’ section validates the feasibility and superiority of our proposed access control model through simulation experiments. Finally, the ‘Conclusion’ section encapsulates the key points of the paper.

## 2 Related work

With the rapid development of medical big data, the vast amount of personal privacy information involved is facing significant threats and challenges. Personal privacy leakage and data misuse have become the core issues affecting medical informatization (13). Blockchain technology and the RAdAC have extensive applications in healthcare. They serve as crucial tools for safeguarding healthcare data integrity, preventing personal privacy breaches, and averting data misuse. In this chapter, we delve into the related work on healthcare access control. This includes discussions on access control based on on-chain storage of Electronic Health Records (EHRs), access control predicated on Interplanetary File System (IPFS) storage of EHRs, and risk-adaptive healthcare access control.

### 2.1 Healthcare access control based on on-chain storage of EHRs

Healthcare access control based on on-chain storage of EHRs involves using blockchain technology to store and encrypt EHRs using cryptographic algorithms. The system then verifies the legitimacy of the user’s identity to make access control decisions. Azaria et al. (14) were pioneers in proposing the use of blockchain technology for access and privilege management of healthcare data. Their system, MedRec, is implemented via three smart contracts: Register Contract (RC), Patient-Provider Relationship Contract (PPR), and Summary Contract (SC). These contracts set and store permission policies and corresponding operations on the blockchain network, replacing the original EHR. Yang et al. (15) enhanced the MedRec framework to protect patient privacy by formulating precise access control policies and secret signatures using Attribute-Based Encryption (ABE). Patel et al. (16) proposed a blockchain-based cross-domain image-sharing framework that uses blockchain as a distributed database to establish radiology studies and patient-defined access permissions. Liu et al. (17) proposed a decentralized access control method based on a proxy re-encryption algorithm. In this method, the healthcare provider stores the patient’s EHR in a private blockchain, and a trusted third party generates the public/private key for the healthcare provider and a re-encryption key based on the requester’s public key. Storing EHRs on a blockchain and using authentication and access control decisions for visitors is a common approach to healthcare access control. However, this approach has some drawbacks; it overlooks the storage limitations of the blockchain, and storing raw or processed EHRs directly on the blockchain significantly increases the storage overhead (18).

### 2.2 Healthcare access control based on IPFS storage of EHRs

The central concept of healthcare access control, based on IPFS for storing EHRs, involves outsourcing EHRs to IPFS or cloud chains, leaving data management to Cloud Service Providers (CSPs) (19, 20). Zhang et al. (21) proposed a blockchain-based architecture for secure clinical data sharing. This architecture stores patients’ sensitive data through CSPs or local databases, and the blockchain only transmits its encrypted reference pointers to securely store and transmit the data. It also develops smart contracts for authentication and access control. Neudecker et al. (22) utilized IPFS as a decentralized cloud storage system to share EHRs by outsourcing the patient’s encrypted medical records to IPFS. Meanwhile, the Ethereum blockchain only stores the corresponding hash values and the owner’s address to manage access. Omar et al. (23) developed an Ethereum-based smart contract framework to address the CT data management problem and outsourced the data to IPFS to reduce storage overhead. Madine et al. (24) extended this approach with a decentralized data management model, enabling patients to take full control of their EHRs in a secure and traceable manner. To address the storage overhead of large-scale EHRs, Xu et al. (25) proposed a blockchain-based hybrid privacy-preserving scheme, Healthchain. This scheme encrypts EHRs using a symmetric encryption algorithm to protect patient privacy and stores them in an IPFS storage system. The corresponding hash values are stored in the blockchain to preserve data integrity in IPFS. While the healthcare access control scheme of storing EHRs in IPFS alleviates the storage overhead of the blockchain to some extent, this scheme has its drawbacks. Despite the traceability of blockchain technology, IPFS is neither secure nor trustworthy, and CSPs may violate patient privacy by snooping on or leaking EHRs (26). Additionally, outsourcing EHRs to IPFS incurs additional cost overhead, increasing the development cost for developers.

### 2.3 Risk-adaptive access control for healthcare

The RAdAC is a centralized access control model that makes decisions through risk assessment results. It offers fine granularity, dynamism, and high flexibility, making it widely applicable in medical big data scenarios (27–30). The accuracy of the risk assessment results, which is crucial to the correctness of the decisions, forms the core of the RAdAC. Wang et al. (31) proposed an access control model for medical big data management. This model uses statistical methods and information theory to calculate the actual offsets of doctors’ work targets and medical record selection, with these offsets serving as the risk of doctors’ access. Hui et al. (32) improved upon the model proposed by Wang et al. They quantified the risk using the EM algorithm and information entropy, and used the average value of the risk of a malicious doctor as the risk threshold, thereby enhancing the accuracy of the risk assessment. Atlam et al. (33) proposed an Adaptive Risk-Based Access Control (AdRBAC) model for IoT that makes authorizations based on household context, resource sensitivity, operation severity, and risk history, and designed a smart contract for monitoring user behavior during an access session. To further improve the accuracy of risk assessment, Jiang et al. (34–37) employed clustering, evolutionary game theory, fuzzy theory, intuitionistic fuzzy trust and regression analysis to quantify or predict the risk of a doctor. Dos Santos et al. (38) proposed an ontology-based approach for risk assessment and a framework based on an extension of XACML for executing risk-based strategies. Despite the flexibility, dynamism, and granularity of the RAdAC, existing research has primarily focused on improving the accuracy of risk assessment results. However, the single point of failure problem due to the centralized authorization of the RAdAC in medical big data scenarios has not been considered.

While blockchain and the RAdAC play significant roles in healthcare privacy protection, existing research in these areas remains limited and presents numerous challenges. Current research on blockchain-based access control in healthcare primarily focuses on the use of authentication and encryption algorithms to prevent unauthorized user access and patient privacy breaches. However, it often overlooks the storage limitations of blockchain and the potential for third-party intrusion into patient privacy through curiosity-driven snooping or EHR leaks. Similarly, research on risk-adaptive-based access control in healthcare has been largely centered on improving decision-making accuracy, often neglecting the single-point-of-failure problem inherent in the RAdAC. To address these issues, we propose storing EHRs in a local database and limiting blockchain access to records and smart contract authorization rules. This approach prevents internal healthcare data leaks without impacting the storage of the HIS or increasing development costs. Additionally, we aim to enhance the traditional RAdAC risk quantification method by integrating risk quantification generated by historical and current behaviors, thereby improving RAdAC decision-making accuracy. Lastly, we plan to deploy the improved RAdAC algorithm into the blockchain in the form of smart contracts to mitigate the single-point-of-failure problem to a certain extent.

## 3 Access control model

### 3.1 Basic framework

In this study, we integrate traditional RadAC with blockchain technology to propose a Smart Contract and Risk-Based Access Control model. The risk associated with a doctor’s choice of medical records is quantified by a risk quantification algorithm within the model. Furthermore, the risk is categorized into current risk and historical risk, with the impact of historical risk represented by a time decay factor. Subsequently, the risk threshold that differentiates honest doctors from malicious ones is calculated by the risk management component of the model. Finally, access control authorization is executed according to the access control contract within the blockchain. Supplementary Figure S1 illustrates the basic architecture of the model, which can be divided into several parts based on its functions:

1. Subject S refers to the staff member who requests access to the HIS, which includes roles such as doctors and nurses. In the model proposed in this paper, we consider the doctor as the accessing subject and focus solely on the risk of privacy leakage that may arise from the doctor’s selection of medical records.
2. Object O refers to the storage system for medical data within the HIS. This system encompasses various types of data, including patient personal information, electronic medical records, disease surveillance data, medical imaging, laboratory test results, and medical research data.
3. Blockchain refers to the distributed ledger that stores the access history of doctors and manages the execution of smart contracts. When a doctor initiates a request to access the HIS storage system, the corresponding smart contract, which is deployed on the blockchain, is automatically executed to authorize access control.
4. Smart Contracts refers to the specific contracts deployed within the blockchain. These include contracts designed for management contract, access history contract and policy contracts.

Despite the multitude of doctors within the system, each one can be uniquely described and identified by a specific set of information. This includes, but is not limited to, a digital signature that uniquely identifies the doctor, as well as their access history. Importantly, the amalgamation of various pieces of information can significantly enhance the authorization rules that describe individual doctors within the model. This, in turn, augments the flexibility and granularity of access control.

### 3.2 Quantification of risk in the model

During a medical consultation, the doctor initially establishes a working objective, typically reflecting their preliminary judgment or definitive diagnosis of the patient’s condition. Subsequently, the doctor selects the medical records pertinent to this working objective for the consultation. If a follow-up visit indicates that the patient does not have the initially diagnosed condition, the doctor resets the working objective and gathers the medical records relevant to the new objective. This iterative process persists until the patient recovers or concludes treatment. Typically, a doctor may request access to multiple medical records due to a common work target. However, doctors with identical work target may not necessarily access the same records, leading to varying levels of risk. Consequently, this study computes the value at risk as the actual deviation in a doctor’s request for access to medical records under a common work target. The value at risk is contingent upon the degree to which the doctor’s request for access to a combination of records fulfills the doctor’s work target.

We assume that honest and malicious doctors follow the following principles: An “honest doctor” is one who adheres to the proper protocols and accesses records only as needed for their work targets. In contrast, a “malicious doctor” is one who may when the objective of the work is determined, attempts to obtain more private information about the user, including attempts to access additional medical records, are considered. This could potentially increase the risk factor associated with the doctor’s access. The risk calculation takes into account these different behaviors to provide a comprehensive assessment.

To quantify the risk of doctors selecting medical records under the same work target, we denote the information of a doctor’s single access behavior as a triple $\langle d_{i},{\mathrm{wt}}_{i},M_{i}\rangle$ , where: $d_{i}\in D$ , with D representing the set of doctors, $wt_{i}\in WT$ , where $wt_{i}$ is the work target chosen by doctor $d_{i}$ , $m_{i}\in M$ , where M denotes the set of medical records. The set of medical records chosen by doctor $d_{i}$ for a given work target wt is denoted as $SM_{d_{i}}^{\mathrm{wt}}$ . Based on the definition of the indicator function, we have got Equation (1) as follows:


(1) $$I\left(x\right)=\{\begin{array}{c} 0,x\neq M\\ 1,x=M \end{array}$$

We assume that the doctor’s wt has been determined, and the number of times doctor $d_{i}$ chooses M in the set $SM_{d_{i}}^{\mathrm{wt}}$ is:


(2) $$f\left(SM_{d_{i}}^{\mathrm{wt}},M\right)=\underset{x\in SM_{d_{i}}^{\mathrm{wt}}}{\sum }I\left(x\right)$$

The main function of Equation (2) is to return the number of times the doctor chooses the same medical record in the same wt .

Similarly, we assume that the doctor’s work target wt has been determined. The probability that the doctor chooses a medical record is then calculated based on this information. This approach allows us to effectively assess the risk associated with each doctor’s selection of medical records.

The doctor’s work target has been determined and the probability that the doctor *d*_i_ chooses the medical record M is *P_di,wt_* (*M*), the formula for *P_di,wt_* (*M*) is shown in Equation (3):


(3) $$P_{d_{i},\mathrm{wt}}\left(M\right)=\frac{f\left(SM_{d_{i}}^{\mathrm{wt}},M\right)}{\sum_{\mathrm{wt}\in WT}f\left(SM_{d_{i}}^{\mathrm{wt}},M\right)}$$

According to the information entropy formula, we can calculate the amount of information that the doctor *d_i_* receives under the work target *wt* as *H_wt_* (*d_i_*), the formula for *H_wt_* (*d_i_*) is shown inEquation (4):


(4) $$H^{\mathrm{wt}}\left(d_{i}\right)=\underset{\mathrm{wt}\in WT}{\sum }P_{d_{i},\mathrm{wt}}\left(M\right)\ln P_{d_{i},\mathrm{wt}}\left(M\right)$$

We used the same methodology to obtain the access history of all doctors under the same work target and calculated the average amount of information for all doctors, which was calculated as shown in Equation (5):


(5) $$H_{\mu }^{\mathrm{wt}}\left(d\right)=\frac{1}{n}\overset{n}{\underset{i=1}{\sum }}H^{\mathrm{wt}}\left(d_{i}\right)$$

By comparing $d_{i}$ with the average amount of information available to doctors, we obtain the value of the risk caused by the choice of medical records under the same work target, which is calculated as shown in Equation (6):


(6) $$\mathrm{Risk}\left(d_{i}\right)=\left\{\left(H^{\mathrm{wt}}\left(d_{i}\right)-H_{\mu }^{\mathrm{wt}}\left(d\right),0\right)\right\}$$

Based on the definitions of honest and malicious doctors, suppose the existence of doctors d_1_ and d_2_. Doctor d_1_ is an honest doctor who will exclusively access medical records related to the specified work target, such as m_1_ and m_2_. On the other hand, doctor d_2_ is a malicious doctor attempting to access a broader range of medical records, including m_1_, m_2_, and m_3_. Assuming an equitable selection of medical records by both doctors, the entropy values computed using formula (4) are 0.693 and 1.099, respectively. Through our risk quantification approach, we found that malicious doctors tend to have a more diverse selection of medical records for a given work objective, resulting in higher entropy values. A higher entropy value signifies a higher risk associated with their access. However, it is challenging to accurately determine whether a doctor is honest based solely on a single access behavior. For instance, a doctor may have had risk values exceeding our set threshold in a certain past period, but the risk value for the current access is below the threshold. If we consider the doctor’s historical and current behavior together, we could classify the doctor as malicious. Yet, if we only judge based on the current behavior, the doctor would be deemed an honest doctor who is permitted system access. This discrepancy arises because the system overlooks the significance of the doctor’s historical behavior, leading to potential misjudgments. Therefore, we propose combining the risk from the doctor’s access history with the current risk to yield a more accurate combined risk assessment. This approach enhances the robustness and reliability of our risk quantification method.

A time decay factor is a factor that, in certain models or contexts, gradually reduces the weight of past data or information in the model over time. The main purpose is to take into account that past data may no longer have the same importance as more recent data. The application of the time decay factor better reflects the novelty and realism of the data and makes the model more relevant to the current situation. To better reflect the importance of doctors’ historical behaviors, we assume that accesses closer to the current time are given more weight, and access further away from the current time are given less weight. Let the time requested by a doctor under a given work target be $T=\left\{t_{1},t_{2},..,t_{k}\right\}$ , where $t_{1}$ is the time of current access, then the corresponding time factor satisfies $\omega_{1}>\omega_{2}>\ldots >\omega_{k}$ . The decay time factor is calculated as shown in Equation (7):


(7) $$\left(t_{j}\right)=\frac{1}{\ln\left(t_{j}+c\right)},1\leq j\leq k$$

where $t_{j}\in T$ , and c is a constant.

Based on the history of doctor’s accesses in the blockchain, we can get the risk generated by the doctor’s first k choices of medical records under the same work target, which can be expressed as Equation (8):


(8) $$Risk_{H}\left(d_{i}\right)=\overset{k}{\underset{j=1}{\sum }}\omega \left(t_{j}\right)Risk_{j}\left(d_{i}\right)$$

Based on the current and historical behavior of the doctor, we obtained the total risk, which can be expressed as Equation (9):


(9) $$\mathrm{TotalRisk}\left(d_{i}\right)=\mathrm{Risk}\left(d_{i}\right)+Risk_{H}\left(d_{i}\right)$$

### 3.3 Risk management in the model

A prevalent strategy for the RadAC involves a management mechanism that utilizes risk thresholds. These thresholds represent the system’s tolerance for the risk each doctor incurs. To enforce access control, the system dynamically updates these risk thresholds. If a doctor’s risk falls below the risk threshold, they are granted continued access. However, if a doctor’s risk surpasses the threshold, their access request is denied until their risk is reduced below the threshold.

The risk threshold is indeed a crucial aspect of risk-adaptive access control, and its appropriateness significantly influences the accuracy of access control. As per the literature (34), the risk threshold is calculated using the average risk of all doctors, encompassing both honest and malicious doctors. However, this calculation method has certain limitations. Specifically, when the proportion of malicious doctors is too large, the risk threshold will increase. Consequently, some malicious doctors may be misclassified as honest doctors, thereby affecting the accuracy of access control. This highlights the need for a more nuanced approach to calculating the risk threshold, one that can effectively differentiate between honest and malicious doctors regardless of their proportion.

In order to prevent the accuracy of access control from being compromised by an over-representation of malicious doctors, we compute the risk thresholds based on the risk of the history of malicious doctors using statistical methods. Denoting $R_{t}\left(d_{i}\right)$ as the risk of doctor $d_{i}$ at time t, we can obtain the risk of all malicious doctors at this stage $R_{t}\left(d\right)=\left\{R_{t}\left(d_{1}\right),R_{t}\left(d_{2}\right),\ldots ,R_{t}\left(d_{n}\right)\right\}$ , then the mean value of the risk of all malicious doctors at that stage can be calculated using Equation (10):


(10) $$R_{t}^{\mu }\left(d\right)=\frac{1}{n}R_{t}\left(d_{i}\right)$$

Using the same method, we can obtain the mean value of risk for all doctors at time t, which can be expressed as Equation (11):


(11) $$R^{\mu }\left(d_{i}\right)=\left\{R_{1}^{\mu }\left(d\right),R_{2}^{\mu }\left(d\right),\ldots ,R_{t}^{\mu }\left(d\right)\right\}$$

Assuming that the risk mean of all doctors in the first t time periods approximately follows a normal distribution, the mean μ and variance σ of this distribution can be obtained, the range of the risk threshold is shown in Equation (12):


(12) $$\varphi \left(t+1\right)=\left[\mu -n\sigma ,\mu +n\sigma \right]$$

where n is chosen depending on the system.

### 3.4 Smart contracts

The auto-execution feature of smart contracts allows us to automate and track certain state transitions in the blockchain. By deploying smart contracts on the blockchain, we can record doctor access history and complete access control authorizations based on historical and current risks. Once the authorization is confirmed through our SCR-BAC in the blockchain, the doctor can access our HIS database off-chain. The SCR-BAC stores the result of this authorization as a transaction in the blockchain, which forms part of the doctor’s access history for their next request. Smart contracts within the SCR-BAC comprises a management contract (MC), an access history contract (AHC), and multiple policy Contracts (PCs). As shown in Supplementary Figure S2, the smart contract within the SCR-BAC is clearly outlined.

#### 3.4.1 Management contract

The role of the MC in the SCR-BAC is to manage the nodes in the system and verify the legitimacy of the doctor’s identity. When a new doctor or database node is added to the system, the MC maps the doctor’s identification string to their address identity (Doc Address or DB Address) and creates a unique SHC for the new node.

#### 3.4.2 Access history contract

The role of the AHC in the SCR-BAC is to maintain the access history of a doctor node, which is created by the MC when the node is added. The AHC stores the Doc Address, the database address (DB Address) of the doctor’s access, the access status of the doctor’s access, the risk value caused by the access, and the address of the applicable PC. When a doctor initiates an access request to the SCR-BAC, the SHC provides a complete list of the access history. This is important because the PC needs to ensure that the doctor’s visit history is real-time and complete.

#### 3.4.3 Policy contract

The role of the PC in the SCR-BAC is to formulate the corresponding access control policy based on the AHC and current behavior. This policy is created by the AHC when a new access record is generated. The AHC stores Doc Address, DB Address, policy, and access control authorization result. When a doctor initiates an access request to the SCR-BAC, the PC formulates the corresponding access control policy based on the AHC and the current behavior. If the doctor’s total risk value is below the threshold, the PC authorizes the access; otherwise, the access is denied.

The PC devises a precise access control policy as shown in Supplementary Figure S3, which illustrates the entire process for authorizing or denying a doctor’s request. It’s important to note that a similar process is implemented for any doctor-initiated request. The PC must calculate the doctor’s historical and current risk values, determine the risk threshold at the current time, and perform the access control authorization. The PC considers the doctor’s behavioral history and the current situation to make the final authorization decision for the access request.

### 3.5 Distributed access control predicated on smart contracts

Traditional RadAC employs centralized authorization, which can lead to issues such as a single point of failure. To address this, we propose an improvement measure that deploys the access control policy in the form of smart contracts into the blockchain. This approach leverages the automatic execution feature of smart contracts to achieve distributed authorization of access control. Specifically, we deploy multiple smart contracts in the blockchain to facilitate the entire access control authorization process. These contracts include MC, AHC and PCs. MC is responsible for managing the doctor nodes in the system and verifying the legitimacy of the doctor’s identity, AHC is responsible for maintaining the access history of the doctor nodes, and PC is responsible for formulating the corresponding access control policies. The entire access control process is illustrated in Supplementary Figure S4. This approach ensures a more robust and reliable access control system by mitigating the risks associated with centralized authorization.

The execution flow of the entire access control contract is described as an algorithm, as shown in Algorithm 1. The process is divided into four steps:

Step-1: Doctor $d_{i}$ initiates an access request after determining the work target. MC gets the identity of the doctor from the blockchain and verifies its legitimacy. If the doctor’s identity is legal, Step-2 is executed; Otherwise, the request is rejected.

Step-2: Firstly, the doctor’s historical risk in the past time t is obtained from the blockchain and the doctor’s historical risk ${\mathrm{Risk}}_{H}\left(d_{i}\right)$ is calculated by combining the time decay factor $\omega \left(t_{j}\right)$ . Secondly, the risk ${\mathrm{Risk}}_{H}\left(d_{i}\right)$ of the doctor’s current behavior is calculated based on the given work objectives and the offset of the doctor’s choice of medical records. Finally, $\mathrm{TotalRisk}\left(d_{i}\right)$ , the combined risk of the doctor, is calculated.

Step-3: The risk of all the malicious doctors in the current time with the same work objective is obtained from the blockchain and the risk threshold is calculated. This operation is repeated until all the risk thresholds in the past time t are calculated. All the risk thresholds in time are taken as a set satisfying normal distribution and its mean μ and variance σ are calculated. Finally, the risk threshold $\varphi \left(t+1\right)$ is calculated.

Step-4: It is determined whether the doctor’s aggregate risk is less than or equal to the set threshold. If it is less than or equal to the risk threshold, authorization is granted; otherwise, authorization is denied and the authorization result is returned.

**Table 1:** 

**Algorithm 1:** Access Control Algorithm.
**Input: A**ccess request: AR **Output:** policy result Get Doctor’s**Identity Information** from Blockchain; **if** identity information is legal**then** **for** each $Risk_{H}\left(d_{i}\right)$ in**Access History do** $Risk_{H}\left(d_{i}\right)=\overset{k}{\underset{j=1}{\sum }}E\left(t_{j}\right)Risk_{j}\left(d_{i}\right)$ ; **end** $\mathrm{Risk}\left(d_{i}\right)=\left\{0,\left(H^{\mathrm{wt}}\left(d_{i}\right)-H_{\mu }^{\mathrm{wt}}\left(d_{i}\right)\right)\right\}$ ; $\mathrm{TotalRisk}\left(d_{i}\right)=Risk_{H}\left(d_{i}\right)+\mathrm{Risk}\left(d_{i}\right)$ ; **for** each $R^{\mu }\left(d\right)$ in**Malicious Doctor’s Access History do** $\varphi \left(t+1\right)=\left[\left(\mu +\sigma \right),\left(\mu +\sigma \right)\right]\text{;}$ **end** **if** $\mathrm{TotalRisk}\left(d_{i}\right)<=\varphi \left(t+1\right)$ **then** policy result = allow; **end** **else** policy result = deny; **end** **else** policy result = deny; **end** **return** policy result;

## 4 Experimental and evaluation

### 4.1 Experimental settings

The data source for the SCR-BAC model proposed in this paper is the National Natural Science Foundation of China (NSFC) project, a joint collaboration between Yunnan University of Finance and Economics and a tertiary hospital in Kunming, China. The experimental data, also obtained from a tertiary hospital in Kunming, China, accounts for a storage space of 1,200G. This data is divided into five databases, with a total of 1,360 data tables, containing 2,139,373 records. The data types include text data, image data, and video data. According to the experimental testing requirements of the model in this paper, we do not need to use all the medical data. Therefore, we only extract a portion of the doctors’ access history from the data for the experiment. We extracted access history information from 50 doctors and used a laptop to deploy an Ethernet private chain to upload the doctors’ access history to the blockchain for evidence. Over time, we calculated the risk value of each doctor based on the access history stored in the blockchain. The doctors’ access history information was obtained as shown in Supplementary Table S1.

### 4.2 Risk quantification experiments and analysis

We simulated and generated access information for 600 doctors based on the access history information of the original 50 doctors. The medical records were labeled using the ICD-10 classification in accordance with healthcare system conventions. Subsequently, risk values were calculated for each doctor.

#### 4.2.1 Results of risk quantification with different number of requests

This section is designed to evaluate the effectiveness of the SCR-BAC model under varying numbers of requests. We simulated the access behavior of 600 doctors, with 90% being honest doctors, 10% being malicious doctors, and there was an over-access rate of 5% by the malicious doctors. The risk meaning for both honest and malicious doctors was calculated separately for each simulation. The results of these experiments are depicted in Supplementary Figure S5.

As depicted in Supplementary Figure S5, the risk quantification method of the SCR-BAC proves to be effective across different numbers of requests. The risk value of malicious doctors is observed to be 9.5–13 times higher than that of honest doctors, demonstrating a clear distinction between the two types of doctors. Furthermore, when the number of requests reaches 15, the impact of increasing the number of accesses on the risk value begins to diminish. Consequently, the risk value associated with a doctor decrease with the number of requests and eventually stabilizes. This indicates that the SCR-BAC model effectively manages risk assessment in varying conditions.

To test the validity of the time decay factor in the SCR-BAC, we compared the mean risk values of 600 doctors with Wang’s (31) scheme. The results of the experiment are shown in Supplementary Figure S6.

As depicted in Supplementary Figure S6, in Wang’s scheme, the risk value of malicious doctors is 6–12 times that of honest doctors. In contrast, in the SCR-BAC model, the risk value of malicious doctors is 9–13 times that of honest doctors. Moreover, the risk values calculated by the SCR-BAC are higher than those in Wang’s scheme for both honest and malicious doctors. This can be attributed to the following reasons: The risk value in the SCR-BAC comprises two components—current risk and historical risk. The risk quantification algorithm of the SCR-BAC introduces historical risk, the influence of which decreases with the weakening of the time decay factor. Therefore, the SCR-BAC exhibits greater precision in distinguishing between the two types of doctors. This demonstrates the effectiveness of incorporating historical data into risk assessment models.

#### 4.2.2 Risk quantification results for different percentages of over accesses

This section is designed to evaluate the effectiveness of the SCR-BAC model under varying percentages of over access. We simulated the access behavior of 600 doctors, with 90% being honest doctors and 10% being malicious doctors. For each work target, the number of access requests is set to 10. The risk meaning for both honest and malicious doctors was calculated separately for each simulation. The results of these experiments are depicted in Supplementary Figure S7. This experiment aims to understand how the SCR-BAC model performs under different conditions of over accesses.

As depicted in Supplementary Figure S7, the risk quantification method of the SCR-BAC proves to be effective across different proportions of over access. The risk value of malicious doctors is observed to be 10–14 times higher than that of honest doctors, demonstrating a clear distinction between the two types of doctors. When the over access proportion reaches 6%, the impact of increasing over access proportion on honest doctors begins to diminish. For malicious doctors, however, the risk value gradually increases as the percentage of over access increases. This is because when the proportion of over access for malicious doctor increases, the gap between their behavior and that of honest doctors widens, making it easier to differentiate between them and determine the risk threshold. This indicates that the SCR-BAC model effectively manages risk assessment in varying conditions of over access.

Similarly, to test the validity of the time decay factor in the SCR-BAC, we compared the mean value of risk of 600 doctors with Wang’s (31) scheme. The results of the experiment are shown in Supplementary Figure S8.

As depicted in Supplementary Figure S8, the risk values calculated by the SCR-BAC are higher than Wang’s scheme for both honest and malicious doctors. The reasons are as follows: the risk value of the SCR-BAC consists of two parts: current risk and historical risk, and the risk quantification algorithm of the SCR-BAC introduces historical risk, and the influence of historical risk decreases with the weakening of the time decay factor. Therefore, the SCR-BAC is more precise in distinguishing between the two types of doctors.

### 4.3 Access control performance and efficiency analysis

In this section, we firstly tested the validity of the SCR-BAC using three metrics: precision, recall, and F_1_, and compared them with Wang’s (31) model. In addition, we analyzed the access control performance of the SCR-BAC and observed the impact on the risk value when the malicious doctor initiates successive access requests. The precision refers to the rate of doctors who are actually malicious among the top K doctors with the highest risk value; the recall refers to the proportion of malicious doctors among the top K doctors with the highest risk to the total number of malicious doctors; and F_1_ refers to the geometric mean of the precision and recall, which is an important metric reflecting the overall performance of the model.

#### 4.3.1 Access control performance with different number of requests

We simulated the access behavior of 600 doctors, with 90% being honest doctors, 10% being malicious doctors, and there was an over-access rate of 5% by the malicious doctors. We test the accuracy, recall and F1 score of the SCR-BAC by adjusting different numbers of access requests. The experimental results are shown in Supplementary Table S2.

As depicted in Supplementary Table S2, the precision of the SCR-BAC is 100% in the top 10 doctors with the highest risk value, even though the precision of the SCR-BAC reaches more than 95% in the top 30 doctors with the highest risk value. As the number of access requests increases, the recall of the SCR-BAC reaches more than 80%, and at least 80% of the 50 malicious doctors are malicious. F_1_ also increases with the number of access requests. This proves that more access information helps the SCR-BAC to accurately learn the access behavior of doctors, more accurately distinguish between two types of doctors and calculate the risk value of doctors. Comparing with Wang’s scheme, the SCR-BAC outperforms Wang’s scheme in Precision, Recall and F_1_ scores regardless of the number of access requests.

#### 4.3.2 Access control performance with different over accesses ratios

We simulated the access behavior of 600 doctors, with 90% of honest doctors and 10% of malicious doctors, and for each job target, the number of access requests is 10. We tested the accuracy, recall, and F_1_ scores of the SCR-BAC by adjusting different proportions of over-access. The experimental results are shown in Supplementary Table S3.

As depicted in Supplementary Table S3, when the over-access ratio is 4% or more, the accuracy of the SCR-BAC reaches more than 90%. When the percentage of over-access is 6%, the recall rate of the SCR-BAC reaches more than 79%. The performance of F_1_ also increases with the increase of the percentage of over-access. Comparing with Wang’s scheme, the SCR-BAC outperforms Wang’s scheme in Precision, Recall and F_1_ scores regardless of the over-access ratio. Because the risk quantification algorithm introduces historical risk, as more malicious behavior makes the gap between the risk values of malicious and honest doctors larger, the SCR-BAC is more likely to distinguish between the two types of doctors.

#### 4.3.3 Access control overall performance analysis

When a doctor initiates an access request, they invariably desire a swift authorization result, regardless of whether the result is an authorization or denial. To verify the efficiency of the access control scheme, we established two conditions for authorization: 1) the combined risk value of the doctor is less than or equal to the risk threshold; 2) the minimum allowable time interval between two consecutive requests is 100 s.

In order to understand the blocking time of the SCR-BAC model with successive initiation of malicious requests and the impact of successive initiation of malicious requests on the real-time risk value of the doctor. We assumed that a malicious doctor initiates a series of malicious requests within a set time, with each request generating a risk of 6.51, exceeding the threshold value of 6.50. After the series of malicious requests ends, the doctor continues to make requests within the set time, with each request generating a risk of 0.5. We then calculated the real-time risk value of the doctor and the blocked time. The results of these experiments are depicted in Supplementary Figure S9.

As illustrated in Supplementary Figure S9, the impact of malicious requests diminishes over time. When a doctor initiates two consecutive malicious requests, the risk value significantly exceeds the threshold value, leading the SCR-BAC to block access for 700 s, a duration longer than that in Wang’s scheme. When the doctor initiates three consecutive malicious requests, the SCR-BAC blocks access for up to 2,200 s, which is substantially longer than the duration in Wang’s scheme.

The reason for this difference lies in the factors considered in the two schemes: In Wang’s scheme, the access control authorization is determined solely by the doctor’s current behavior, without taking into account the doctor’s historical behavior. In contrast, the SCR-BAC determines access control authorization based on both the doctor’s current behavior and historical behavior. When a malicious behavior generates a high risk value, the impact generated by this high risk value does not immediately dissipate. Instead, it takes a certain period of time for this impact to diminish and be disregarded. Therefore, the access control efficiency of the SCR-BAC is significantly higher than that of Wang’s scheme, demonstrating the importance of considering both current and historical behaviors in access control authorization. This highlights the effectiveness of the SCR-BAC model in managing risk assessment under varying conditions and behaviors.

## 5 Conclusion

Medical big data holds significant value in medical research and patient treatment. However, there is a risk of internal privacy leakage when doctors access this data. In this paper, we propose a medical big data access control model based on smart contracts and risk. This model quantifies the risk value based on both the current and historical behaviors of doctors. It describes the impact of historical behaviors through a time decay factor and deploys the access control policy into the blockchain in the form of a smart contract. This approach addresses the single point of failure problem inherent in traditional RadAC. Simulation results indicate that the access control model proposed in this paper effectively limits the access behavior of malicious doctors, thereby mitigating the risk of internal abuse and privacy leakage in medical big data. This demonstrates the potential of our model in enhancing the security and privacy of medical big data access.

## Data availability statement

The original contributions presented in the study are included in the article/Supplementary material, further inquiries can be directed to the corresponding author/s.

## Author contributions

XP: Conceptualization, Data curation, Software, Writing – original draft. RJ: Funding acquisition, Investigation, Supervision, Writing – review & editing. ZS: Funding acquisition, Investigation, Supervision, Writing – review & editing. ZL: Funding acquisition, Supervision, Writing – review & editing. LY: Conceptualization, Data curation, Software, Writing – original draft.
