# Supplementary material for: A medical big data access control model based on smart contracts and risk in the blockchain environment
Source: Front Public Health. 2024 Mar 28;12:1358184. doi: 10.3389/fpubh.2024.1358184 (PMC11007037; doi:10.3389/fpubh.2024.1358184)
Supplement: Supplementary file 1 [file Data_Sheet_1.docx]

Supplementary Material

# Supplementary Figures and Tables

All the figures and tables involved in this study are as follows:

## Supplementary Figures

**Supplementary Figure 1**. Access control system architecture based on smart contracts and risk


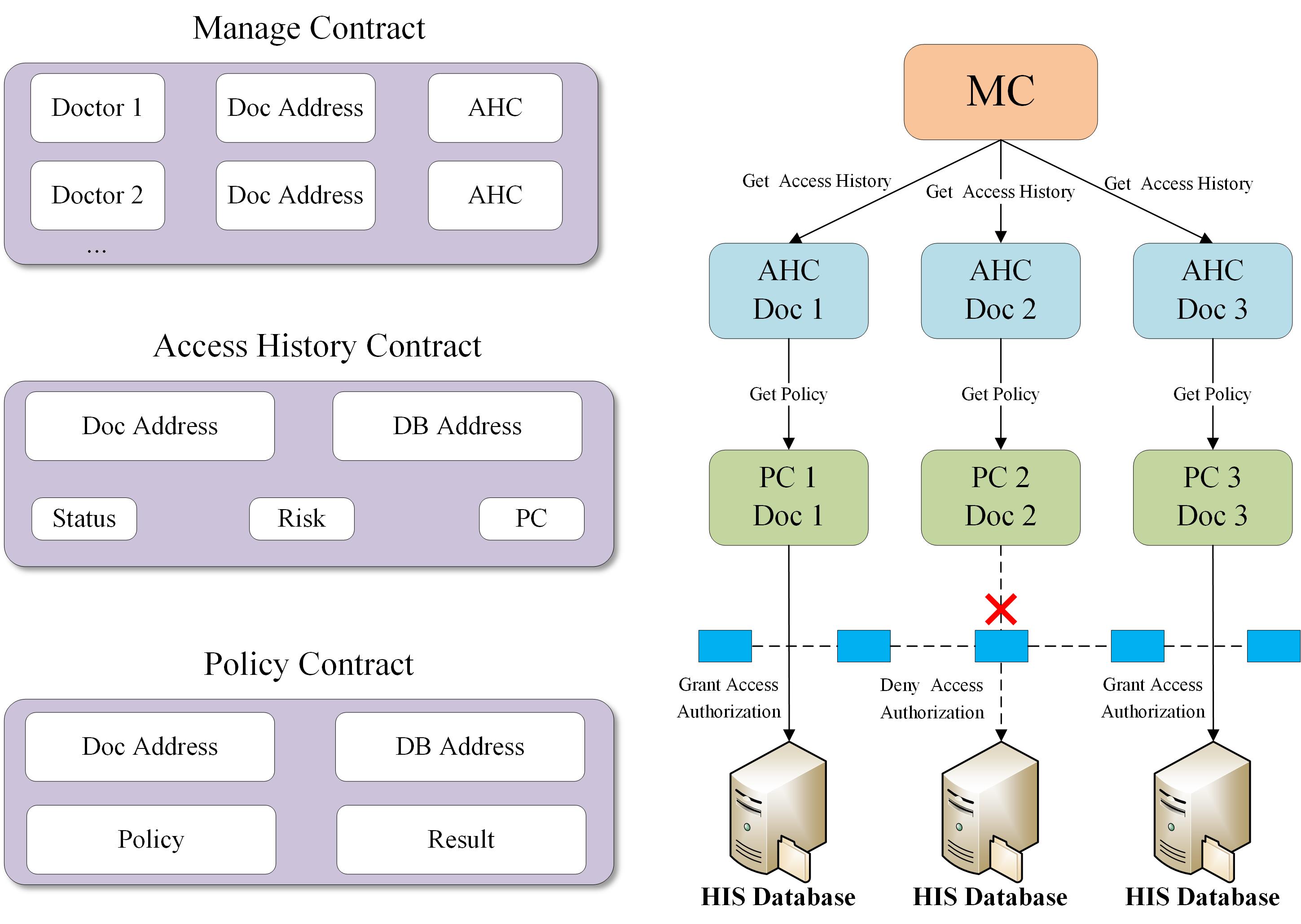


**Supplementary Figure 2**. SCR-BAC smart contracts on the blockchain


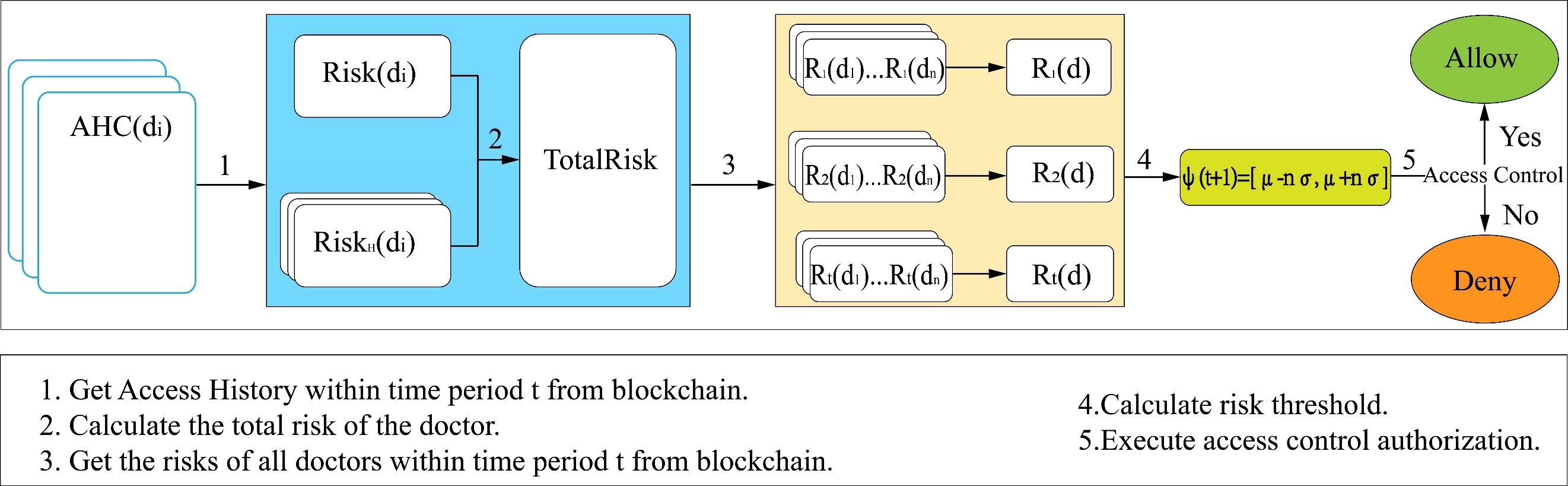


**Supplementary Figure 3**. The execution flow of the PC


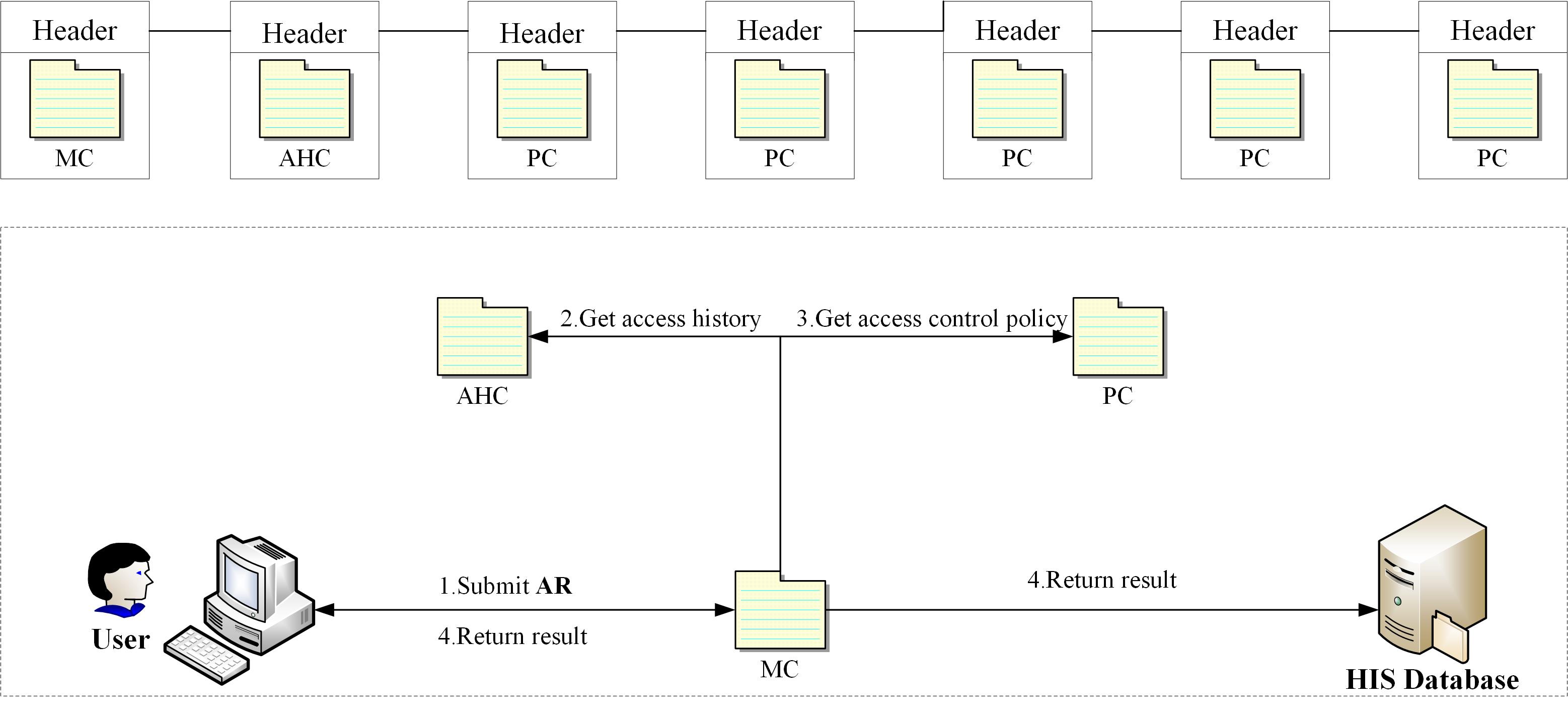


**Supplementary Figure 4**. Access control contracts in blockchain


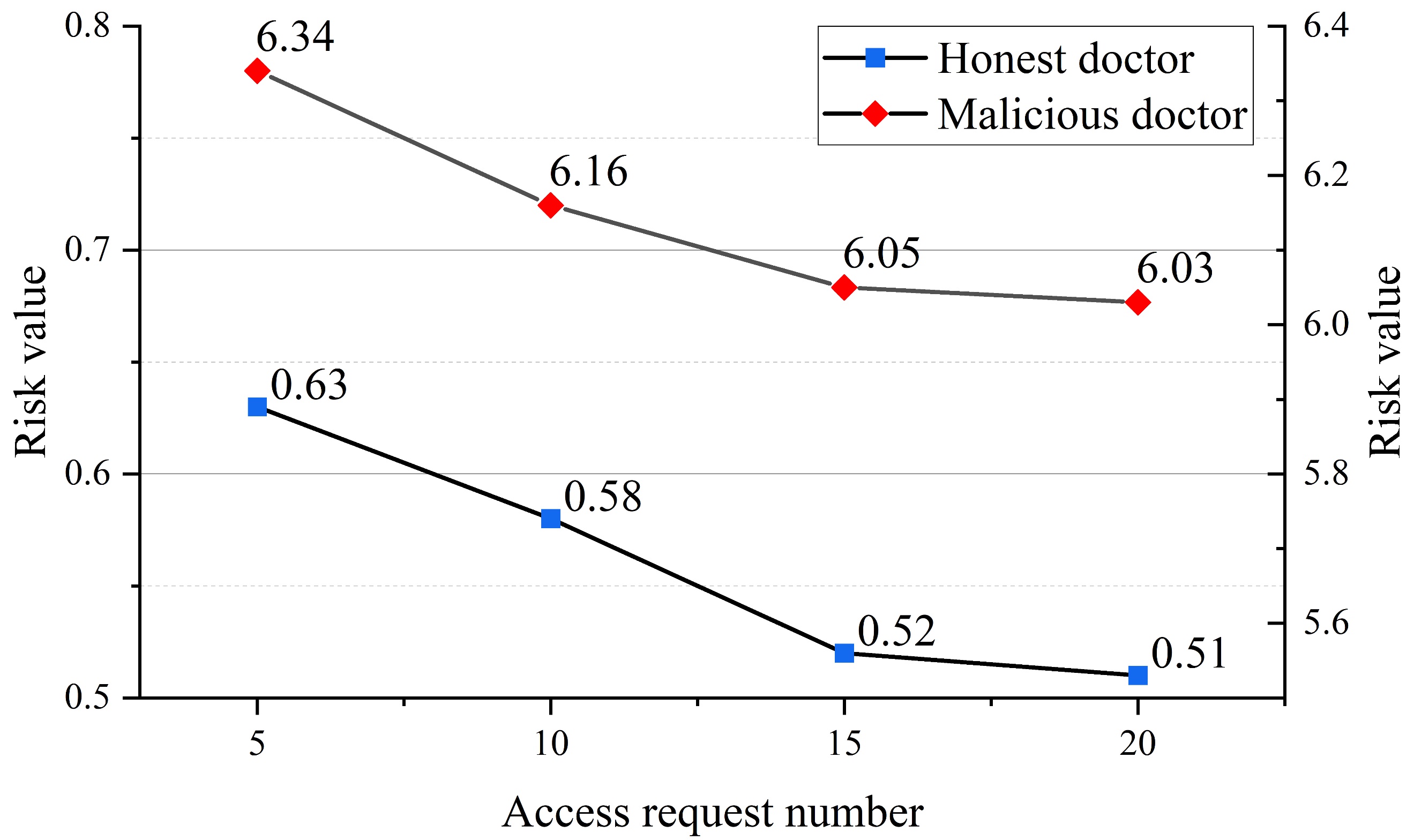


**Supplementary Figure 5**. Results of risk quantification with different number of requests


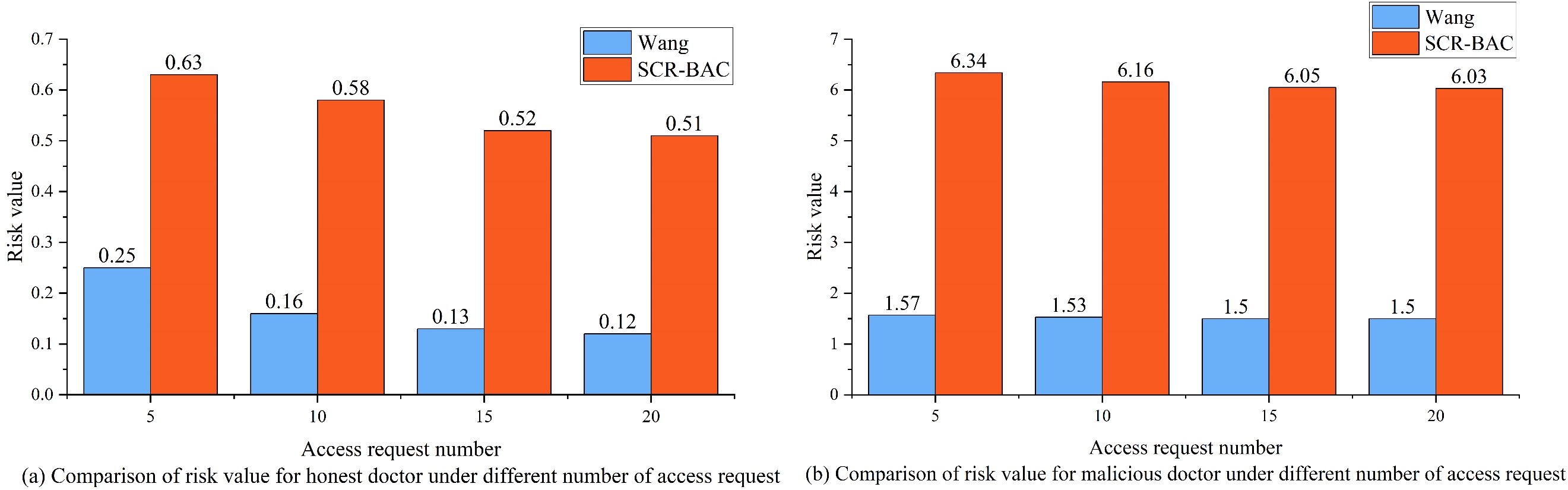


**Supplementary Figure 6**. Comparison of Value at Risk for Different Number of Requests


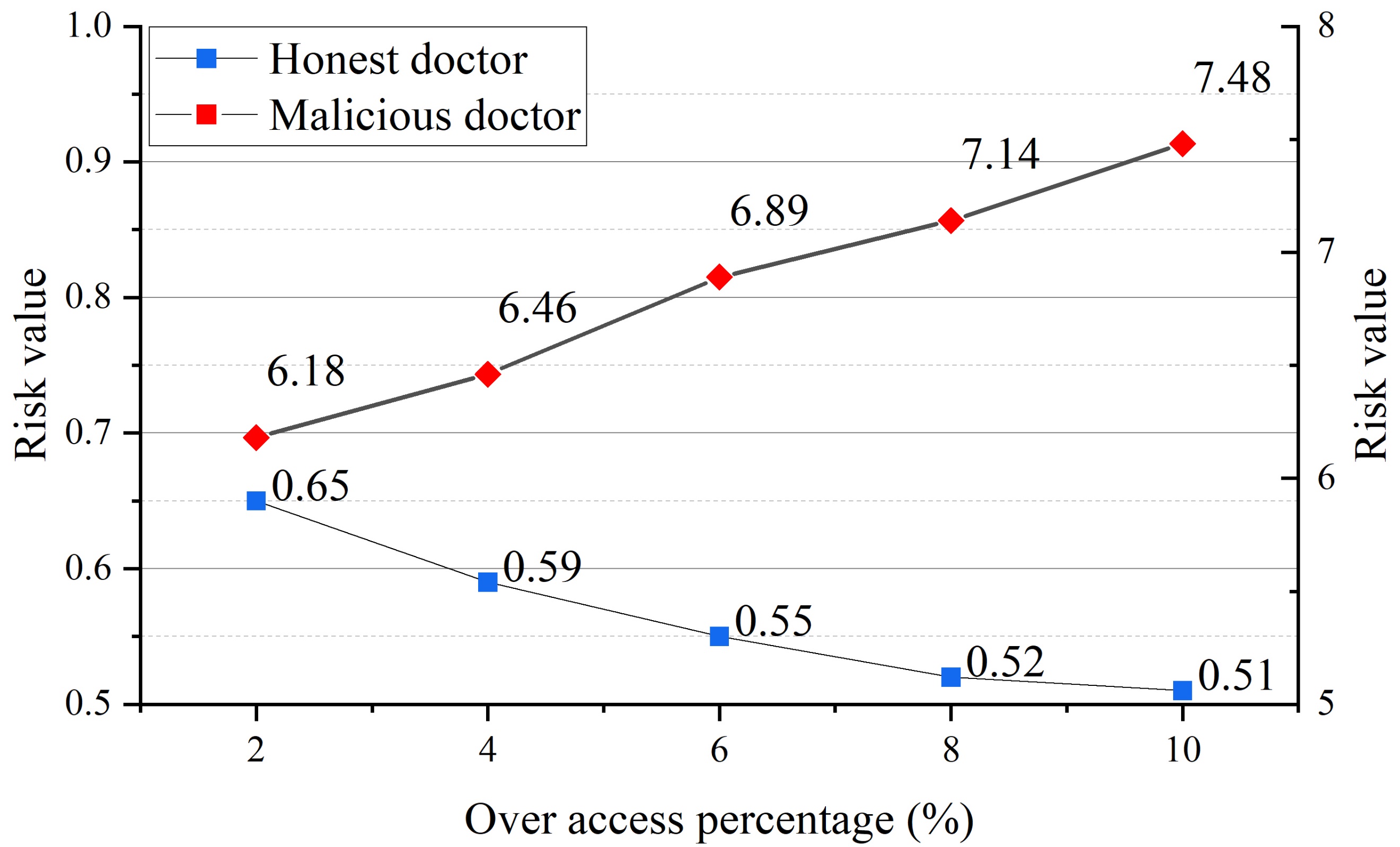


**Supplementary Figure 7**. Comparison of value at risk for different percentages of over access


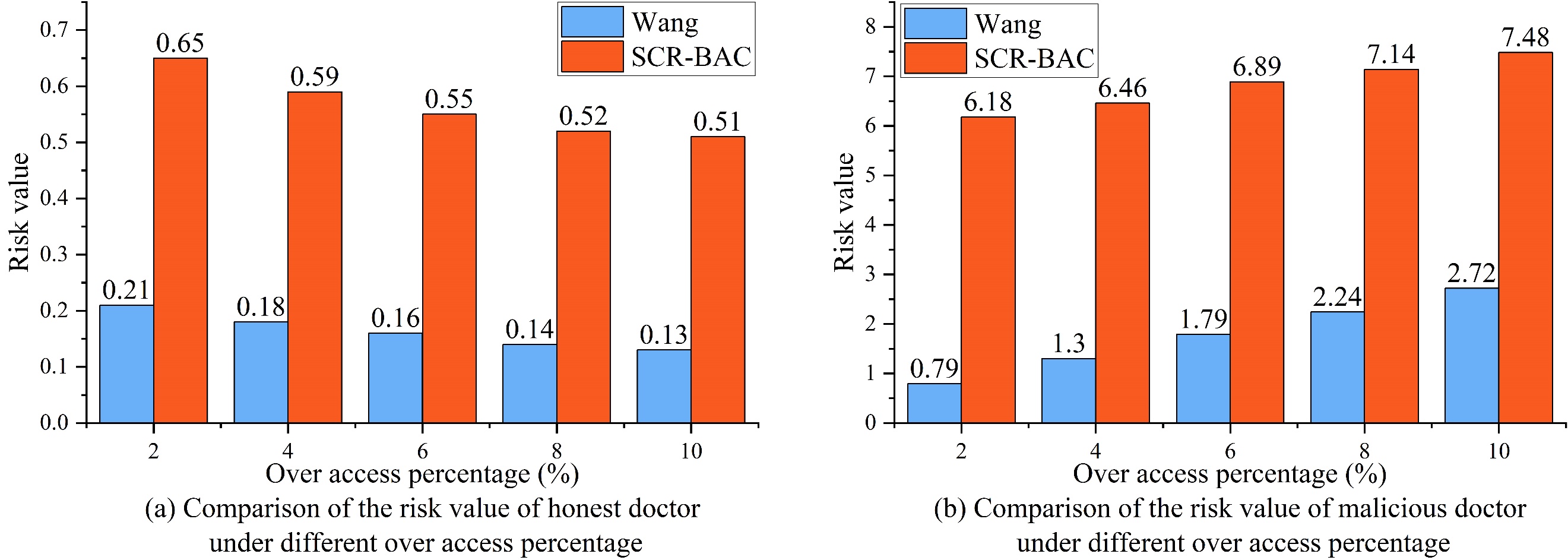


**Supplementary Figure 8**. Risk quantification results for different percentages of over accesses


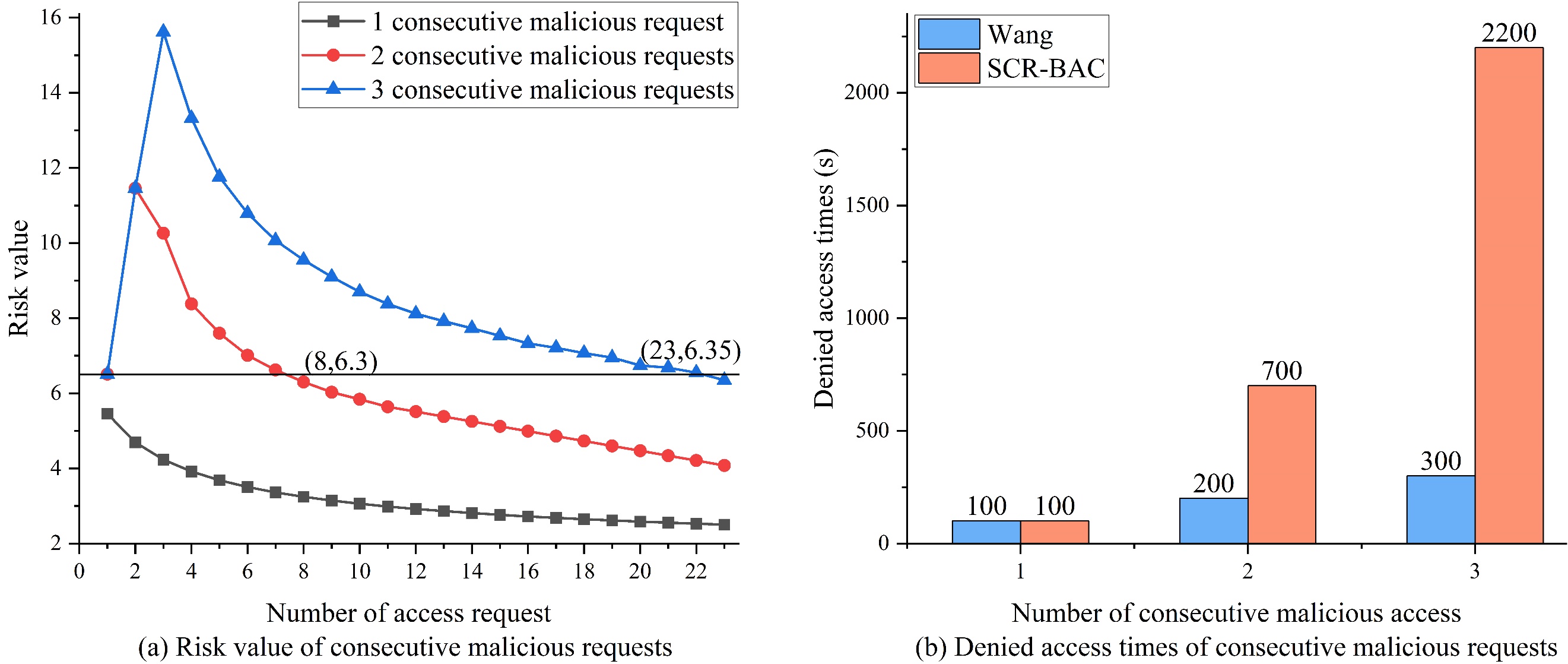


**Supplementary Figure 9**. SCR-BAC efficiency analysis

## Supplementary Tables

| **Doc Address** | **DB Address** | **Status** | **Risk** |
| --- | --- | --- | --- |
| 0xb51f6d86d4c763531056a517543568fbafc32a98 | 0x3f23c7b929bfed419lef6064ffcb25972eald925 | Honest access | 0.58 |
| 0xc1d5f73d4e825143865b637585671ccabd85b43 | 0xab735c486496354523bf47aea78df7110ae495 | Honest access | 0.61 |
| 0xca35c1f847753da548ecf7056dd2c33f7ac7354d | 0xb87f6d98d4c856641214a146874214adefa832 | Over access | 6.54 |
| … | … | … | … |
| 0xd335c1f847753da548ecf70156dd2c33f7ac754c | 0xb875c7b45bfed855ad6064aafc35671afed873 | Honest access | 0.53 |

**Supplementary Table 1**. Information on the access history of different doctors

| **The number of access requests** | **x** | **Precision** | | **Recall** | | **F_1_** | |
| --- | --- | --- | --- | --- | --- | --- | --- |
|  |  | **SCR-BAC** | **Wang** | **SCR-BAC** | **Wang** | **SCR-BAC** | **Wang** |
| 5 | 10 | 1.00 | 1.00 | 0.19 | 0.19 | 0.32 | 0.32 |
|  | 20 | 0.97 | 0.95 | 0.36 | 0.35 | 0.53 | 0.51 |
|  | 30 | 0.95 | 0.87 | 0.54 | 0.48 | 0.65 | 0.62 |
|  | 40 | 0.86 | 0.80 | 0.63 | 0.59 | 0.69 | 0.68 |
|  | 50 | 0.8 | 0.70 | 0.74 | 0.65 | 0.71 | 0.67 |
| 10 | 10 | 1.00 | 1.00 | 0.19 | 0.19 | 0.32 | 0.32 |
|  | 20 | 1.00 | 1.00 | 0.39 | 0.38 | 0.56 | 0.55 |
|  | 30 | 0.97 | 0.97 | 0.55 | 0.55 | 0.7 | 0.7 |
|  | 40 | 0.81 | 0.78 | 0.64 | 0.58 | 0.69 | 0.67 |
|  | 50 | 0.79 | 0.74 | 0.80 | 0.70 | 0.77 | 0.72 |
| 15 | 10 | 1.00 | 1.00 | 0.19 | 0.19 | 0.32 | 0.32 |
|  | 20 | 1.00 | 1.00 | 0.39 | 0.37 | 0.55 | 0.54 |
|  | 30 | 1.00 | 0.97 | 0.56 | 0.52 | 0.76 | 0.69 |
|  | 40 | 0.93 | 0.88 | 0.65 | 0.65 | 0.79 | 0.75 |
|  | 50 | 0.84 | 0.76 | 0.81 | 0.70 | 0.82 | 0.73 |
| 20 | 10 | 1.00 | 1.00 | 0.19 | 0.19 | 0.32 | 0.32 |
|  | 20 | 1.00 | 1.00 | 0.39 | 0.37 | 0.58 | 0.54 |
|  | 30 | 1.00 | 0.93 | 0.56 | 0.52 | 0.76 | 0.67 |
|  | 40 | 1.00 | 0.85 | 0.67 | 0.63 | 0.79 | 0.72 |
|  | 50 | 0.88 | 0.80 | 0.83 | 0.74 | 0.85 | 0.77 |

**Supplementary Table 2**. Performance of SCR-BAC with different number of requests

| **Percentage** | **x** | **Precision** | | **Recall** | | **F_1_** | |
| --- | --- | --- | --- | --- | --- | --- | --- |
|  |  | **SCR-BAC** | **Wang** | **SCR-BAC** | **Wang** | **SCR-BAC** | **Wang** |
| 2% | 10 | 0.70 | 0.70 | 0.13 | 0.13 | 0.22 | 0.22 |
|  | 20 | 0.68 | 0.65 | 0.28 | 0.24 | 0.38 | 0.35 |
|  | 30 | 0.65 | 0.63 | 0.42 | 0.35 | 0.50 | 0.45 |
|  | 40 | 0.60 | 0.60 | 0.44 | 0.44 | 0.51 | 0.51 |
|  | 50 | 0.56 | 0.52 | 0.54 | 0.49 | 0.53 | 0.50 |
| 4% | 10 | 1.00 | 1.00 | 0.19 | 0.19 | 0.32 | 0.32 |
|  | 20 | 1.00 | 0.95 | 0.38 | 0.35 | 0.51 | 0.51 |
|  | 30 | 0.98 | 0.87 | 0.49 | 0.48 | 0.65 | 0.62 |
|  | 40 | 0.92 | 0.72 | 0.57 | 0.54 | 0.73 | 0.62 |
|  | 50 | 0.90 | 0.72 | 0.71 | 0.67 | 0.78 | 0.69 |
| 6% | 10 | 1.00 | 1.00 | 0.19 | 0.19 | 0.32 | 0.32 |
|  | 20 | 1.00 | 1.00 | 0.37 | 0.37 | 0.54 | 0.54 |
|  | 30 | 1.00 | 0.97 | 0.57 | 0.54 | 0.71 | 0.69 |
|  | 40 | 0.98 | 0.92 | 0.63 | 0.56 | 0.81 | 0.79 |
|  | 50 | 0.92 | 0.90 | 0.79 | 0.56 | 0.87 | 0.86 |
| 8% | 10 | 1.00 | 1.00 | 0.19 | 0.19 | 0.32 | 0.32 |
|  | 20 | 1.00 | 1.00 | 0.39 | 0.37 | 0.58 | 0.54 |
|  | 30 | 1.00 | 1.00 | 0.56 | 0.56 | 0.76 | 0.72 |
|  | 40 | 1.00 | 0.98 | 0.78 | 0.72 | 0.84 | 0.83 |
|  | 50 | 1.00 | 0.92 | 0.89 | 0.85 | 0.90 | 0.88 |
| 10% | 10 | 1.00 | 1.00 | 0.19 | 0.19 | 0.32 | 0.32 |
|  | 20 | 1.00 | 1.00 | 0.37 | 0.37 | 0.54 | 0.54 |
|  | 30 | 1.00 | 1.00 | 0.56 | 0.56 | 0.72 | 0.72 |
|  | 40 | 1.00 | 0.98 | 0.80 | 0.72 | 0.85 | 0.83 |
|  | 50 | 1.00 | 0.92 | 0.90 | 0.85 | 0.90 | 0.88 |

**Supplementary Table 3**. Performance of SCR-BAC with different number of over accesses
